# Atopic Dermatitis and Major Depressive Disorder: is there causality?

**DOI:** 10.1192/j.eurpsy.2023.1754

**Published:** 2023-07-19

**Authors:** C. S. Reis, A. Vieira, P. S. Martins

**Affiliations:** Psychiatry and Mental Health, University Hospital Center of São João, Porto, Portugal

## Abstract

**Introduction:**

The association between Atopic Dermatitis (AD) and Major Depressive Disorder (MDD) has long been reported by some population-based observational studies. However, observational studies are susceptible to potential confounders and inverse causation, rendering it difficult to conclude about the causality of such association. Mendelian randomization (MR) analysis is a novel epidemiological method to assess the causation between an exposure and an outcome, with less susceptibility to potential confounders and reverse causation by using genetic variants as instrumental variables.

**Objectives:**

To report a clinical case of depression in association with atopic dermatitis and to review what contributions MR studies have been bringing to the matter of causality between AD and MDD.

**Methods:**

Case report and literature review based on PubMed using the terms “atopic dermatitis”, “eczema”, “depression", “depressive”, “mood” and “Mendelian randomization”, which were searched in the title and abstract fields.

**Results:**

Case-report: A 26-year-old man was admitted for inpatient treatment with a clinical picture of sadness, irritability, social isolation and insomnia, with 4 months of evolution, aggravated by suicidal ideation in the preceding days. On examination of the mental status, the patient had a frankly depressed mood, with congruent affects. He was contemplating suicide methods, pointing to sodium nitrite intoxication as an option. The patient related these symptoms to the worsening of his atopic dermatitis. In fact, he had a history of other depressive episodes contemporaries with periods of dermatological worsening.

Literature review: The PubMed research identified 7 articles, 4 of which assessed the causal effect of AD on MDD. Three studies did support a causal effect of AD genetic risk on MDD. One study supported a small causal effect of AD on MDD, with the significance disappearing when a stricter threshold for selection of single-nucleotide polymorphisms was applied.

**Conclusions:**

The MR studies included in this poster favour the absence of a causal effect of AD on MDD, suggesting that the comorbidity observed clinically is unlikely to be causal. We must be aware that these studies are few and are not free of limitations (e.g. subgroup analysis for age and severity was not carried out, AD and MDD diagnosis were self-reported in some cases). Further research may help clarify the existence of causality and/or uncover the factors responsible for the observed association of AD with MDD in observational studies.

**Disclosure of Interest:**

None Declared

